# Supplementary material for: MetaQTL: a package of new computational methods for the meta-analysis of QTL mapping experiments
Source: BMC Bioinformatics. 2007 Feb 8;8:49. doi: 10.1186/1471-2105-8-49 (PMC1808479; doi:10.1186/1471-2105-8-49)
Supplement: Additional File 7 — MetaQTL Package : jar file and tutorial. This Zip archive contains both the MetaQTL JAR file and the files of the tutorial. [file 1471-2105-8-49-S7.zip › org.inra.metaqtl/doc/org/metaqtl/graph/ChromAxe.html]

ChromAxe


|  |  |  |
| --- | --- | --- |
| **Overview** \| **Package** \| **Class** \| **Use** \| **Tree** \| **Deprecated** \| **Index** \| **Help** | |  |
| **PREV CLASS**   **NEXT CLASS** | **FRAMES**    **NO FRAMES**     **All Classes** |  |
| SUMMARY: NESTED \| FIELD \| CONSTR \| METHOD | DETAIL: FIELD \| CONSTR \| METHOD |  |


---


## org.metaqtl.graph Class ChromAxe

```
java.lang.Object
  org.metaqtl.graph.ChromAxe
```

---

``` public class ChromAxe extends java.lang.Object ```

---

**Constructor Summary**

`ChromAxe(double yMin,
double yMax,
double length)`


| **Method Summary** | |
| --- | --- |
| `double` | `getHeight()` |
| `double` | `getX()` |
| `double` | `getYMax()` |
| `double` | `getYMin()` |
| `void` | `scale(double sy)`             Scale the axe in y. |
| `double` | `scaleHeight(double h)` |
| `double` | `scaleY(double d)` |
| `void` | `setX(double x)` |
| `void` | `setYMax(double max)` |
| `void` | `setYMin(double min)` |
| `double` | `transformY(double d)`             Do a affine transformation of the given position `d` which is assumed to be in [0,dmax] in order to have its representation in the chromosome axe. |

**Methods inherited from class java.lang.Object**

`clone, equals, finalize, getClass, hashCode, notify, notifyAll, toString, wait, wait, wait`

**Constructor Detail**

### ChromAxe

```
public ChromAxe(double yMin,
                double yMax,
                double length)
```


**Method Detail**

### getYMax

```
public double getYMax()
```

:   **Returns:**: Returns the yMax.

---


### setYMax

```
public void setYMax(double max)
```

:   **Parameters:**: `max` - The yMax to set.

---


### getYMin

```
public double getYMin()
```

:   **Returns:**: Returns the yMin.

---


### setYMin

```
public void setYMin(double min)
```

:   **Parameters:**: `min` - The yMin to set.

---


### getHeight

```
public double getHeight()
```

:   **Returns:**

---


### transformY

```
public double transformY(double d)
```

:   Do a affine transformation of the given
    position `d` which is assumed
    to be in [0,dmax] in order to have its
    representation in the chromosome axe.

    :   **Parameters:**: `d` - **Returns:**

---


### scaleY

```
public double scaleY(double d)
```

---


### scaleHeight

```
public double scaleHeight(double h)
```

:   **Parameters:**: `d` - **Returns:**

---


### scale

```
public void scale(double sy)
```

:   Scale the axe in y.

    :   **Parameters:**: `sy` -

---


### getX

```
public double getX()
```

:   **Returns:**: Returns the x.

---


### setX

```
public void setX(double x)
```

:   **Parameters:**: `x` - The x to set.


---


|  |  |  |
| --- | --- | --- |
| **Overview** \| **Package** \| **Class** \| **Use** \| **Tree** \| **Deprecated** \| **Index** \| **Help** | |  |
| **PREV CLASS**   **NEXT CLASS** | **FRAMES**    **NO FRAMES**     **All Classes** |  |
| SUMMARY: NESTED \| FIELD \| CONSTR \| METHOD | DETAIL: FIELD \| CONSTR \| METHOD |  |


---
